# Electrophysiological Evidence of Space‐Number Associations in 9‐Month‐Old Infants

**DOI:** 10.1111/cdev.13584

**Published:** 2021-05-24

**Authors:** Roberta Bettoni, Margaret Addabbo, Hermann Bulf, Viola Macchi Cassia

**Affiliations:** ^1^ University of Milano‐Bicocca; ^2^ Milan Center for Neuroscience

## Abstract

Infant research is providing accumulating evidence that number‐space mappings appear early in development. Here, a Posner cueing paradigm was used to investigate the neural mechanisms underpinning the attentional bias induced by nonsymbolic numerical cues in 9‐month‐old infants (*N* = 32). Event‐related potentials and saccadic reaction time were measured to the onset of a peripheral target flashing right after the offset of a centered small or large numerical cue, with the location of the target being either congruent or incongruent with the number’s relative position on a left‐to‐right oriented representational continuum. Results indicated that the cueing effect induced by numbers on infants’ orienting of eye gaze brings about sensory facilitation in processing visual information at the cued location.

There is extensive literature showing that, in adults, mental representation of ordered numerical magnitudes is embedded into represented space, as humans think numbers as spatially organized along a horizontal continuum. In Western readers, such continuum is oriented from left to right, with small numbers associated to left, and large numbers to right. These pervasive associations are referred to in the literature as Spatial‐Numerical Associations (SNAs). One of the most well‐documented evidence of this phenomenon is the so‐called Spatial‐Numerical Association of Response Codes (SNARC) effect (Dehaene, Bossini, & Giraux, 1993), by which smaller numbers (e.g., 1 or 2) are responded faster with the left hand and large numbers (e.g., 8 or 9) with the right hand. Such response pattern suggests a compatibility effect between the left and right sides of one's own body and a left‐to‐right oriented numerical representation, which would be automatically activated when perceiving a number.

The link between numbers and space is also supported by several behavioral studies in adults showing that the processing of numerical information brings about the activation of spatial attention (e.g., Dodd, Van der Stigchel, Leghari, Fung, & Kingstone, 2008; Fischer, Castel, Dodd, & Pratt, 2003; but see Fattorini, Pinto, Rotondaro, & Doricchi, 2015). For instance, in the seminal study by Fischer et al. (2003), the perception of small/large centrally presented Arabic digits (i.e., 1 or 2 vs. 8 or 9) speeded up the detection of left/right‐lateralized targets depending upon the number's magnitude: Small numbers facilitated left target detection, whereas large numbers facilitated right target detection. In the same vein, eye gaze responses were faster to the left visual hemifield in response to central small numerical cues (i.e., 2 dots) and faster to the right hemifield in response to large numerical cues (i.e., 9 dots; Bulf, Macchi Cassia, & de Hevia, 2014; Fischer, Warlop, Hill, & Fias, 2004).

This so‐called attentional SNARC effect is of particular importance because it indicates that the automatically triggered covert shift of selective attention over the internal representation of numbers influences the allocation of selective attention in the external space, suggesting the recruiting of shared parietal neural circuitries (see van Dijck, Abrahamse, Acar, Ketels, & Fias, 2014). The existence of direct interfacing between internal and external selective attention is also supported by evidence suggesting that overlearned sensorimotor and attentional habits acquired through reading and writing practices modulate the directional aspects of the internal spatial representation of number in adults (Göbel, Shaki, & Fischer, 2011). As an example, the classical SNARC effect is absent or even reversed in cultures who write/read from right to left (e.g., Shaki, Fischer, & Petrusic, 2009). Similarly, a brief exposure to left‐to‐right or right‐to‐left reading sessions influences the direction of finger counting behavior in preliterate children (Göbel, McCrink, Fischer, & Shaki, 2018). These findings are interpreted as evidence that the spatial representation of number results from an individual's mental strategy to spatially organize information in working memory during task execution (van Dijck & Fias, 2011) according to the dominant direction of their cultural environment. In fact, despite the wealth of evidence supporting the role of cultural artifacts in shaping the directionality of the spatial representation of number, developmental research is providing accumulating evidence that SNAs are apparent in infancy, well before the acquisition of language, reading/writing, and counting practices (Bulf, de Hevia, & Macchi Cassia, 2016; de Hevia, Girelli, Addabbo, & Macchi Cassia, 2014).

By measuring visual looking times toward laterally presented stimuli, two recent studies showed that 0‐ to 3‐day‐old human neonates preferred to look at either the left or right sides of the screen depending on the magnitude of visual quantities (Di Giorgio et al., 2019) or auditory quantities paired with visual figures (de Hevia, Veggiotti, Streri, & Bonn, 2017). These results suggest that newborns perceive a numerical stimulus as more or less salient depending on the spatial position to which it is associated: smaller numbers (e.g., 4, 6) to the left, and larger numbers (e.g., 18, 36) to the right side of space. These spontaneous associations of quantities to different spatial positions are akin to the SNARC effect observed in adults (e.g., Wood, Willmes, Nuerk, & Fischer, 2008), children (e.g., de Hevia & Spelke, 2009; Patro & Haman, 2012; van Galen & Reitsma, 2008), and even nonhuman animals (e.g., Rugani, Vallortigara, Priftis, & Regolin, 2015), supporting the hypothesis that SNAs represent a universal cognitive strategy originating from biological constraints inherent to the developing human brain.

More direct evidence for interactions between spatial attention and the coding of nonsymbolic numerical information—that is, the attentional SNARC effect—in infancy comes from an eye‐tracking study conducted with 8‐month‐old infants (Bulf et al., 2016), whose results replicated those obtained by Fischer et al. (2003) in adult participants. Time‐to‐target fixations were recorded while infants oriented their gaze toward peripheral targets appearing right after the onset of a centered small‐magnitude or large‐magnitude nonsymbolic cue—that is, a set of 2 or 9 dots. Like the adults (Bulf et al., 2014; Fias, Lammertyn, Reynvoet, Dupont, & Orban, 2003), infants were faster at fixating targets appearing on the right when cued by large numbers and targets appearing on the left when cued by small numbers. These findings suggest that the perception of nonsymbolic numbers affects visuospatial processing during the first year of life. The facilitation of time‐to‐target fixations when the peripheral target was presented at a location that is congruent with the number's relative position along a left‐to‐right oriented horizontal SNA indicates a shift of attention to the peripheral location and increased efficiency of processing at that attended location.

Although the recording of time‐to‐target fixation under free‐looking conditions is a valid tool to assess visual attention mechanisms in infancy (e.g., Bulf & Valenza, 2013), an investigation of the neural mechanisms that underpin the attentional bias induced by numerical cues in infants is missing. This study aims to fill this gap.

In adults, neural correlates of the processing bias induced by symbolic numerical cues on the perception of lateralized targets have been explored using event‐related potentials (ERPs). Results showed that numbers generated a modulation of the early sensory P1 and the later cognitive P3 components evoked by the peripheral targets across parietal sites, whose amplitude was affected by the congruency between number size and target location (Salillas, El Yagoubi, & Semenza, 2008; Schuller, Hoffmann, Goffaux, & Schiltz, 2015). The sensory P1 component is typically enhanced when a peripheral target is presented at an attended location that has been previously cued by a peripheral stimulus (e.g., Hillyard & Anllo‐Vento, 1998). Therefore, the finding of a larger P1 for valid (e.g., small number‐cues followed by left‐lateralized targets) than invalid trials (e.g., small number‐cues followed by right‐lateralized targets) in the aforementioned studies provides neural evidence that a centrally presented numerical cue can influence the orienting of attention to peripheral locations.

Electrophysiological studies have shown that in infants as well, covert shifts of attention to a peripheral cued location can be indexed by ERP responses to a subsequent target. When tested with a spatial cueing procedure, infants as young as 4, 5 months of age show an enhancement of the early P1 component over occipital electrodes for targets presented at the same location (valid trials) of the cues compared to those appearing at a location that was not previously cued (invalid or neutral trials; e.g., Richards, 2000, 2001, 2005). In these studies, the P1 validity effect resulted from the cue‐target spatial relation, as both the cue and the target were presented at a peripheral location. However, more recently two studies with 6‐month‐old infants have shown that ERP correlates of visuospatial attention to peripheral targets can also be modulated by central directional social cues, such as hand grasping gestures (Natale et al., 2017) or point‐light displays of a human walker (Lunghi, Di Giorgio, Benavides‐Varela, & Simion, 2020; Lunghi, Piccardi, Richards, & Simion, 2019). In these studies, ERP responses to peripheral target objects were modulated by the congruent versus incongruent relation between the directionality (leftward or rightward) of the dynamic social cue and the position of the target object. In particular, Lunghi et al. (2019) reported a larger P1 amplitude over occipital‐parietal sites in response to peripheral targets appearing at locations congruent with the walking direction of the point‐light displays, indicating the enhancement of information processing at the attended location induced by the central cue.

In light of this evidence, this study aimed to probe the neural correlates of the number‐induced attentional cueing effect observed in previous eye‐tracking studies with infants (Bulf et al., 2016) by recording target‐locked ERP responses within a cueing paradigm similar to that used by Natale et al. (2017) and Lunghi et al. (2020, 2019). Infants were presented with a large or small nonsymbolic numerical array (i.e., 2 or 9, as in Bulf et al., 2016), which acted as a central cue, and measured ERP responses to the onset of a target object subsequently flashed at left or right peripheral location. Because the aim was to investigate if the attentional cueing effect indexed by infants' eye movements in previous studies brings about sensory facilitation in processing visual information at the cued location, infants of the same age as in Bulf et al. (2016) were tested. It is hypothesized to observe modulations of the early P1 component in occipital areas in response to the target onset. In particular, we expected to observe a larger P1 amplitude when targets appearing on the right are cued by a large number and targets appearing on the left are cued by a small number (valid condition), compared to when the cue‐target relation is reversed (invalid condition). Furthermore, it is expected to observe a validity effect in infants' latency of eye gaze shifts toward the peripheral targets, which should be signaled by more correct eye gaze responses and/or faster saccadic response times to valid trials relative to invalid trials.

## Method

### Participants

The sample size for the ERP analyses was based on an a‐priori Power Analysis for a repeated‐measures analysis of variance (ANOVA) using the effect size reported in a previous study (Lunghi et al., 2019) for 6‐month‐old infants experiencing attentional cueing effects in a similar design ($\eta_{p}^{2}$ = .031), which revealed that 19 participants should lead to an 85% chance to observe a significant effect with an alpha level of .05. Forty‐two infants were invited to participate in the study. Data from 10 infants were excluded due to fussiness resulting in failure to complete testing. Behavioral analyses were then performed on 32 infants (16 females, *M*_age_ = 285.75 days, *SE* = 9.74, range = 269–306 days). Data from an additional 13 infants were discarded to meet inclusion criteria for the ERP analyses: Eight infants were excluded due to uninterpretable electroencephalogram (EEG) data resulting from no completion of at least eight trials per validity condition (see de Klerk, Johnson, & Southgate, 2015; Lunghi et al., 2019, 2020 for a similar approach), and five were excluded due to excessive movement artifacts. Therefore, ERP analyses were performed on a subsample of 19 infants (9 females, *M*_age_ = 285 days, *SE* = 2.70, range = 269–306 days). The attrition rate for the ERP analyses (55%) is similar to other EEG studies with infants about this age (e.g., de Klerk et al., 2015; Stets, Stahl, & Reid, 2012). Participants were recruited via a written invitation that was sent to parents based on birth records provided by neighboring cities; parents gave their written informed consent. The protocol was carried out in accordance with the ethical standards of the Declaration of Helsinki and approved by the Ethical Committee of the University of Milano Bicocca.

### Stimuli, Apparatus and Procedure

**Figure 1:**
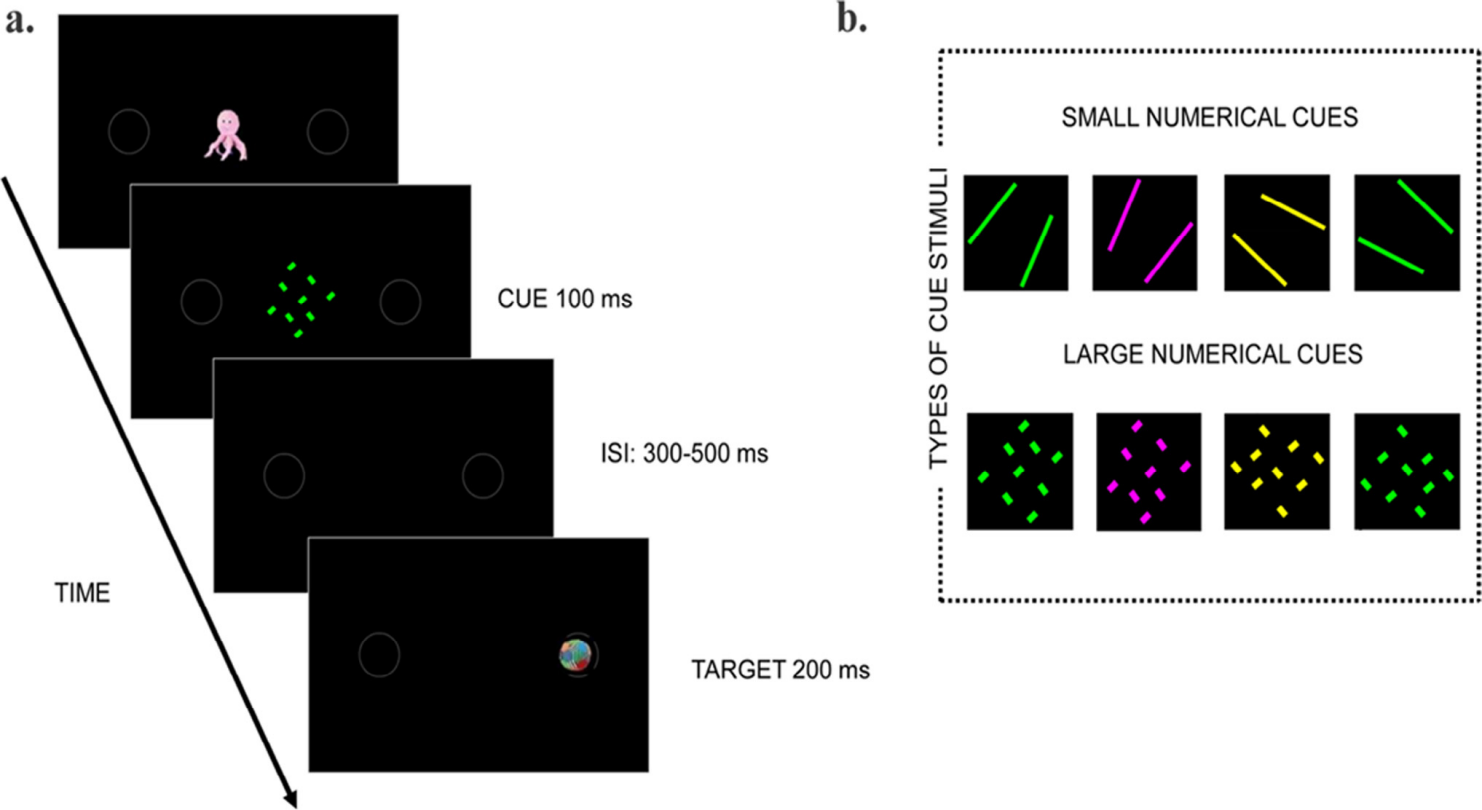
Trial structure and exemplar stimuli. Examples of (a) a valid trial, in which a right‐sided target is cued by a large numerical array, and (b) the two‐item and the nine‐item arrays used as numerical cues.

Testing took place in an electrically shielded dark cabin. Infants were seated on the parent's lap at approximately 60 cm from a 24‐inch monitor. A video camera installed above the screen recorded the infants' eye and body movements for offline coding. Stimuli were presented using E‐prime 2.0 software (Psychology Software Tools, Inc., Pittsburgh, PA, USA). To focus infant's attention on the differences between the two numerical magnitudes that would be subsequently presented during the cueing task (i.e., 2 and 9), prior to the EEG testing session, they were administered two loops of twelve 4 sec‐trials alternating arrays of two or nine animated characters, for a total presentation time of 96 s. Subsequent experimental trials then began, each starting with a central animated fixation point flanked by two peripheral grey circle‐shaped outlines (6° of visual angle, 11° from the center of the screen) that appeared on a black background. As soon as the experimenter judged that the infant was looking at the fixation point, they started the trial by replacing the fixation point with a numerical cue that remained on the screen for 300 ms. After a variable delay (range: 300–500 ms) from the cue offset a target stimulus appeared for 200 ms within one of the two peripheral circles, at a valid spatial location (i.e., left‐sided for the small cue and right‐sided for the large cue) or invalid (i.e., left‐sided for the large cue and right‐sided for the small cue) with the magnitude of the numerical cue (Figure 1).

The numerical cue consisted of two‐item or a nine‐item arrays, that were matched for the cumulative area occupied by the items (~8 cm^2^) and their total contour length (~34.5 cm), as well as for the size of the virtual square subtended by the items (~52 cm^2^). The rectangular‐shaped items composing the two‐item and the nine‐item arrays measured, respectively, 0.5 × 8.1 cm and 0.73 × 1.2 cm; they were arranged randomly within the virtual square, with their shorter side aligned with the horizontal plane. Each numerosity (i.e., 2 or 9) could appear in three different colors (yellow, green, and pink) and four orientations (two leftward, and two rightward), created by rotating the array to the right by 90° three times, which resulted in 12 different arrays per numerosity (Figure 1). On each trial, the displayed numerical cue was selected randomly from the pool of 12 arrays, and the displayed target stimulus was selected randomly from a pool of sixteen different images of a static colorful ball (1.75 cm in radius) varying in color (Figure 1). Trials were administered in blocks, each block composed of eight valid trials (i.e., left‐sided target preceded by a two‐item cue, and right‐sided target preceded by a nine‐item cue) and eight invalid trials (i.e., left‐sided target preceded by a nine‐item cue, and right‐sided target preceded by a two‐item cue). Valid and invalid trials appeared randomly, and cue numerosity and target position were equally distributed within each block. There was no restriction on the number of trial blocks presented to each participant: The experimental session terminated when the infant had looked away from the screen during five consecutive trials.

### ERP Recording and Analysis

**Figure 2:**
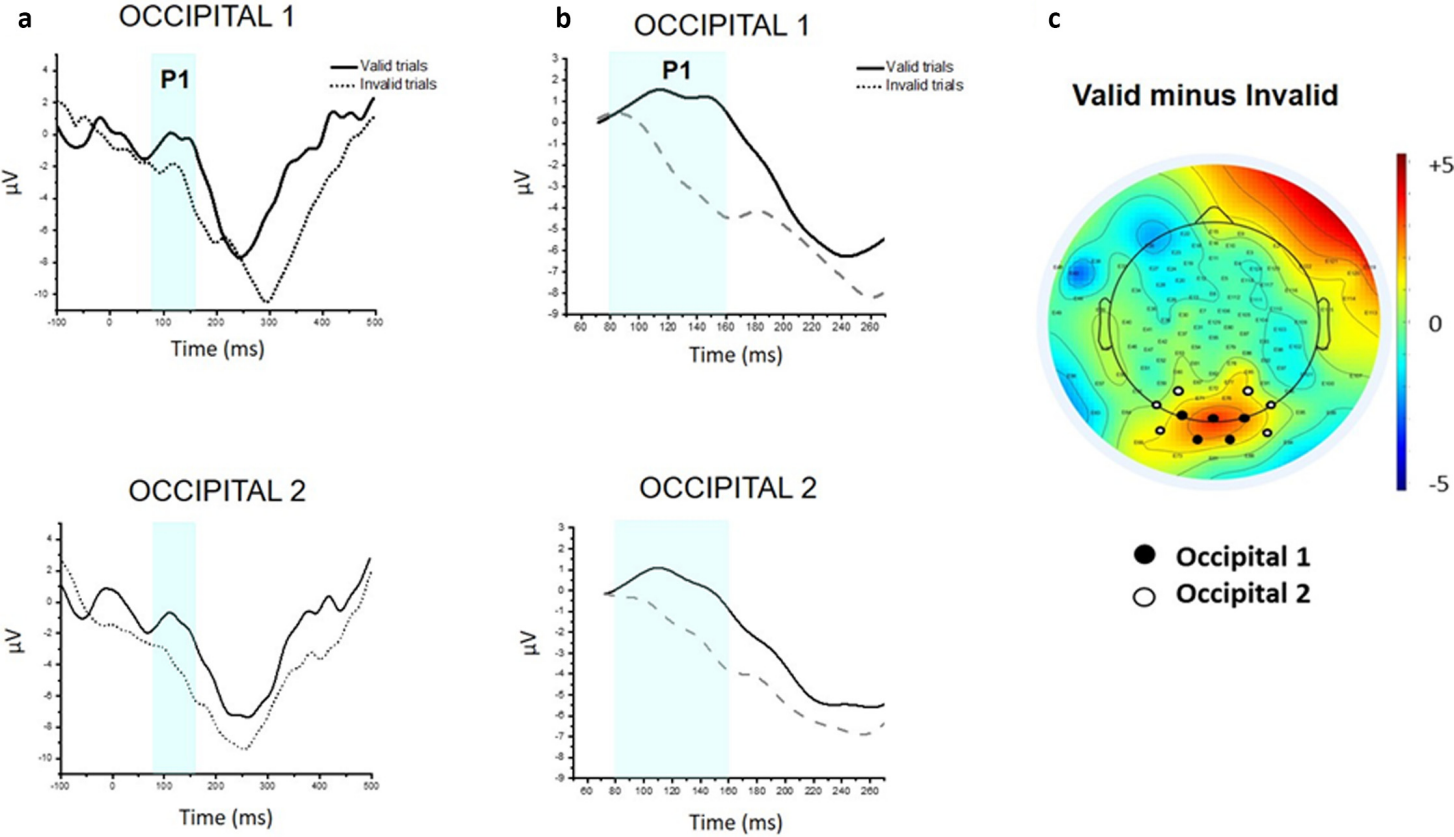
(a) Grand‐averaged event‐related potential waveforms of the P1 (80–160 ms) component for valid (solid line) and invalid trials (dashed line) in the Occipital 1 and Occipital 2 clusters. (b) P1 waveforms for valid and invalid trials plotted as differences from the preceding negative trough (50–70 ms). (c) Topographical scalp potential difference map illustrating the P1 validity effect (Valid minus Invalid) at the selected clusters (Occipital 1 and Occipital 2).

Continuous scalp EEG was recorded from 128 scalp sites using a dense array EGI recording system (Electrical Geodesic, Eugene, OR) connected to a NetAmps 300 amplifier (Electrical Geodesic). The vertex electrode (Cz) was used as a single online reference, and signals were sampled at 500 Hz. Channels impedance was checked before signal recording and considered acceptable if lower than 50 kΩ. EEG data were preprocessed offline using NetStation 4.5 (Electrical Geodesic). The continuous EEG signal was segmented to 1,300 ms post‐stimulus onset, with a baseline period beginning 100 ms prior to target onset. Data segments were bandpass filtered at 0.3–30 Hz and baseline‐corrected using mean voltage during the 100 ms pre‐stimulus period. To eliminate bad EEG epochs containing artifacts two procedures were applied to the segmented data. First, automated artifact detection allowed to detect of individual sensors that showed >200 μV voltage changes within the segment period, and the entire trial was excluded if more than 18 bad channels (15%) were detected. Second, visual inspection of the data led to the rejection of trials in which additional artifacts linked to eye‐movements, eye‐blinks, and other body movements were observed. Additionally, segments in which the infant did not look at the cue or did not keep central fixation at least until the target offset were identified by offline coding of the infant's eye movements and were marked as bad segments. Specifically, bad segments, including trials in which infants' gaze was not aligned with the center of the screen at cue offset or correct gaze shifts started 200 ms before the target offset, were excluded from the analysis. Therefore, the whole trial time‐window comprised between the cue offset and the target offset (200 ms post‐target onset) was free of eye‐blinks and eye‐movements. Of the remaining trials, individual channels containing artifacts were interpolated using a spherical spline. Similar to other infant visual ERP studies (Brooker et al., 2019; Lunghi et al., 2019, 2020), an inclusion criterion of eight artifact‐free trials per condition (i.e., valid and invalid trials) was adopted to include participants in the final sample. A similar number of trials contributed to the final analysis for the valid and invalid condition (*M* = 14.05, range = 9–22 vs. *M* = 13.26, range = 8–24), *t*(18) = 1.385, *p* = .183, *d* = .171, and, within each condition, trials were evenly distributed across target position, and hence across numerical cues (valid‐left: *M* = 7.21, *SD* = 2.46 vs. valid‐right: *M* = 6.84, *SD* = 2.50, *t*(18) = 0.597, *p* = .558, *d* = .149; invalid‐left: *M* = 6.79, *SD* = 2.68 vs. invalid‐right: *M* = 6.47, *SD* = 2.84, *t*(18) = 0.590, *p* = .563, *d* = .116). For each participant, separate grand‐average waveforms were generated for valid and invalid trials by collapsing left‐sided and right‐sided targets. Based on previous studies (Lunghi et al., 2019; Natale et al., 2017; Xie & Richards, 2017) and visual inspection of scalp topography, 12 occipital electrodes were identified for the ERP analysis: electrodes 70, 74, 75 (Occipital 1) and 65, 66, 69 (Occipital 2) over the left hemisphere to analyze ERPs in response to valid and invalid right‐sided targets, and electrodes 75, 82, 83 (Occipital 1) and 84, 89, 90 (Occipital 2) to analyze ERPs in response to valid and invalid targets (Figure 2). The mean amplitude of the P1 was extracted within a time window of 80–160 ms, which was chosen based on previous studies (Lunghi et al., 2019, 2020; Richards, 2000) and visual inspection of the data. The P1 amplitude was corrected for the pre‐negative deflection by using the mean minus trough procedure (Xie & Richards, 2017; Figure 2). This correction controls for the differences at the pre‐ P1 negative amplitude and reduces the effects of negative trends which could drive the differences in the adjacent P1 component (Peykarjou, Pauen, & Hoehl, 2014; Xie & Richards, 2017). The mean minus trough correction was conducted by measuring the pre‐ P1 negative amplitude (C1), defined as the mean activity between 50–70 ms. C1 mean amplitude was then subtracted from the P1 mean amplitude (80–160 ms time window) for each condition. A repeated‐measure ANOVA was performed on mean amplitude with Validity (valid vs. invalid) and Electrode Cluster (Occipital 1 vs. Occipital 2) as within‐subjects factors.

### Gaze Behavior Recording and Analysis

Infants' gaze behavior was coded offline frame‐by‐frame at the nearest 40 ms. A coder blind to the experimental hypothesis recorded, for each participant, the accuracy of saccadic responses, that is the percentage of trials in which the infant shifted their gaze toward the target, and saccadic reaction times (SRTs) for correct saccades, that is the time elapsed from the onset of the target and the onset of the infant's gaze shift. Uncorrected gaze shifts (shifts toward the spatial position opposite to where the target appeared), gaze shifts that started before the target appeared, and shifts that did not start from the center of the screen were discarded. SRTs longer than 1,000 ms and shorter than 100 ms were marked as, respectively, delayed and anticipatory saccades, and were therefore excluded from the analysis (see Bulf & Valenza, 2013; Lunghi et al., 2020). Moreover, for each participant, SRTs shorter and longer than 3 *SD* from the participant's mean for each condition were excluded.

A similar number of trials contributed to the calculation of saccade accuracy and SRTs for the valid and invalid condition in both the entire infant sample (*M* = 14.03 vs. *M* = 13.37), *t*(31) = 0.969, *p* = .340, and the subsample of infants included in the ERP analysis, (*M* = 15.95 vs. *M* = 15.47), *t*(18) = 0.567, *p* = .578, *d* = .116. Saccade accuracy and SRTs were compared across valid and invalid trials through paired‐sample *t*‐tests.

## Results

### Target‐Locked P1 ERP Component

The 2 (Validity) × 2 (Electrode Cluster) ANOVA performed on corrected P1 mean amplitude revealed a main effect of Validity, *F*(1, 18) = 4.828, *p* = .041, $\eta_{p}^{2}$ = .212, as the P1 component in response to the target was larger on valid trails (*M* = 0.763 μV, *SE* = 0.991 μV) than on invalid trials (*M* = −1.700 μV, *SE* = 1.016 μV). Not the Electrode Cluster main effect, *F*(1, 18) = 0.202, *p* = .659, $\eta_{p}^{2}$ = .011, nor the Validity × Electrode Cluster interaction, *F*(1, 18) = 1.776, *p* = .199, $\eta_{p}^{2}$ = .090, attained significance (Figure 2).

### Saccade Accuracy and SRTs

**Figure 3:**
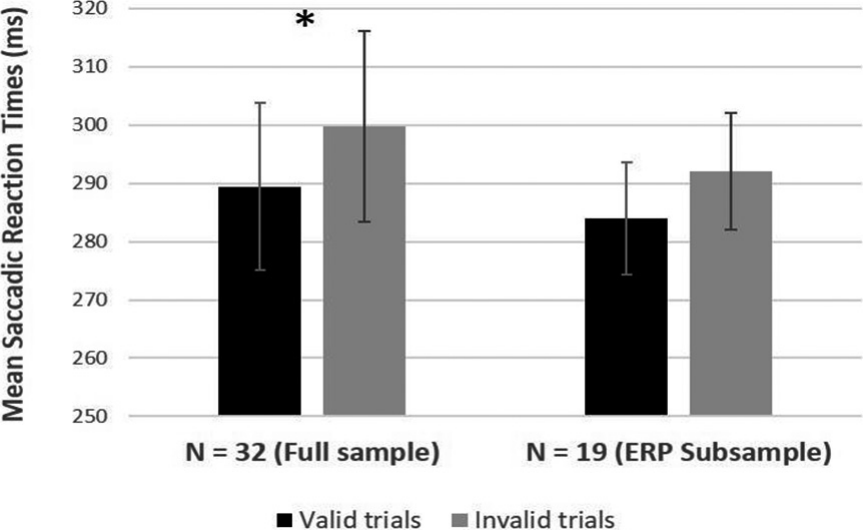
Saccadic reaction times (SRT). Mean SRTs for correct saccades toward the target on valid and invalid trials for the entire infant sample, and for the subsample of infants included in the event‐related potential (ERP) analysis. *p = .052.

Analyses of saccade accuracy and SRTs data were performed on both the entire sample of infants tested (*N* = 32), and the subsample of infants included in the ERP analysis (*N* = 19). Infants in the entire sample performed a correct saccade on 89.75% of the trials (range = 72–100), and a paired‐sample *t‐*test revealed no difference in saccade accuracy between valid (*M* = 89.53%, *SE* = 1.56) and invalid (*M* = 90.44%, *SE* = 1.83) trials, *t*(31) = 0.435, *p* = .666, *d* = .094. The *t*‐test performed on SRTs showed that infants were marginally faster in shifting their gaze to the target on valid trials (*M* = 289.44 ms, *SE* = 14.37) than invalid trials (*M* = 299.78 ms, *SE* = 16.31), *t*(31) = 2.019, *p* = .052, *d* = .119 (Figure 3).

For the smaller subsample of infants included in the ERP analysis, saccade accuracy was 88.74% (range = 72–100), with no difference between valid (*M* = 89.26%, *SE* = 2.33) and invalid (*M* = 88.63%, *SE* = 2.38) trials, *t*(18) = 0.209, *p* = .837, *d* = .061. Similarly, no difference between valid (*M* = 283.97 ms, *SE* = 9.63) and invalid trials (*M* = 292.03 ms, *SE* = 10.80) was evident for SRTs, *t*(18) = 1.062, *p* = .302, *d* = .181 (Figure 3).

## Discussion

This study aimed to explore the neural correlates of attentional shifts induced by numerical magnitudes in infancy. To this aim, target‐locked ERP responses were recorded within a cueing paradigm similar to that used in previous ERP studies with adults (e.g., Salillas et al., 2008; Schuller et al., 2015) and infants (e.g., Lunghi et al., 2019, 2020; Natale et al., 2017).

Nine‐month‐old infants were exposed to central nonsymbolic numerical cues, that is, a 2‐dots array or a 9‐dots array (as in Bulf et al., 2016). Their ERP responses to targets that subsequently appeared at the left or right spatial location were measured. Results showed an enhancement of the early occipital P1 component in response to targets presented at positions that were congruent with the magnitude of the cue (i.e., left‐lateralized targets cued by 2‐dots arrays and right‐lateralized targets cued by 9‐dots arrays) with respect to targets appearing at positions incongruent with the cue magnitude (i.e., left‐lateralized targets cued by 9‐dots arrays and right‐lateralized targets cued by 2‐dots arrays).

This finding replicates and extends earlier demonstrations of a validity effect at the level of the occipital P1 in response to peripheral targets appearing soon after a peripheral cue (Richards, 2005), or a dynamic central cue whose movement is spatially informative with respect to the target position (Lunghi et al., 2019, 2020; Natale et al., 2017). Critically, the current results extend these earlier demonstrations by showing that even spatially noninformative central cues are capable of orient infants' visual attention toward the left or right side of space. They demonstrate, for the first time in infancy, a sensory facilitation induced by numerical cues on the perception of spatially lateralized targets, suggesting that, as previously shown in adults (Schuller et al., 2015), the effect of numerical information on visuospatial attention emerges at early stages of sensory processing. Indeed, as first proposed by Fischer et al. (2003), the validity effect observed in the current data appears to be due to the mere encoding of the numerical information embedded in the cue: by activating infants' representation of numerical magnitude, the encoding of the small versus large numerical cue prompted a covert orientating of attention over the internal oriented representational space upon which number is mapped, and this, in turn, produced enhanced sensory processing at the cued location in the external space.

The results also converge with the previous report that numbers affect infants' eye gaze behavior in the external space (Bulf et al., 2016), as SRTs data from the whole sample of infants tested in the study were marginally faster for valid than invalid trials. Although the comparison was far from being significant for the subsample of infants included in the ERP analysis, they also showed a similar trend in the SRTs data, supporting an association between the oculomotor behavior subtending visuospatial orienting of attention and processing facilitation of information at the cued location in infants (see also Lunghi et al., 2019, 2020), similar to what is reported in adults (e.g., Schuller et al., 2015). The failure to replicate a fully significant validity effect in SRTs, similar to that reported in the eye‐tracking study by Bulf et al. (2016), likely results from methodological differences between the current and previous studies. Specifically, target presentation time was contingent upon infant's behavior in Bulf et al.’s (2016) study, as target offset occurred as soon as the infant looked at it for at least 100 ms. In contrast, because the current project aimed to record infant's target‐locked ERP responses, target presentation duration was fixed, and rather short, in this study (i.e., 200 ms), and this has likely limited the rewarding nature of the task and increased its difficulty for the infants. Moreover, the poor temporal resolution of the offline frame‐by‐frame coding of gaze shifts in this study, as opposed to the online‐automated coding in the Bulf et al.’s (2016) study, may have reduced the ability to capture fine‐grained differences in infants' oculomotor behavior across conditions.

It is worth noting that non‐numerical continuous variables were controlled in this study by keeping cumulative surface area, contour length, and the size of the virtual area subtended by the items constant across numerical arrays. Nonetheless, because not all continuous variables that covary with the number can be controlled simultaneously, the 2‐dots and the 9‐dots numerical arrays varied for item density. This implies that the possibility exists that the cueing effect observed in the current data was driven by variations in this non‐numerical continuous variable that covaried with the number, rather than numerical information per se. This hypothesis could be rejected in light of previous eye‐tracking evidence that surface area—that is, a continuous magnitude variable—did not act as a cue in orienting infants' visual attention toward peripheral regions of space in the same way as number did (Bulf et al., 2016). In light of this, we shall conclude that number was critical in driving the validity effect observed in infants' ERP responses and gaze behavior in this study. Future research shall investigate this further by testing whether the number‐induced attentional effect is maintained when all non‐numerical variables are made noninformative, for example, by varying each of them randomly across small and large numerical arrays.

Overall, the finding that shifts of visuospatial attention induced by numerosities in preverbal infants are accompanied by enhanced neural processing at the attended locations closely resembles ERP evidence with symbolic numbers in adults (Schuller et al., 2015), and points to the presence of a common neural mechanism underlying SNAs across the life span. The formation of SNAs constitutes a critical milestone in the development of mathematical skills, as the development of a precise spatial representation of numbers is crucial for the understanding of the principle of ordinality of numbers. Indeed, an automated access to a spatial representation of numbers is impaired in children with developmental dyscalculia (e.g., Piazza et al., 2010), whose brain activation and performance in numerical tasks has been shown to improve significantly following training programs focused on the linear spatial representation of numbers (Kucian et al., 2011). The current demonstration of enhanced neural processing of peripheral stimuli appearing at locations congruent with the relative position of the preceding numerical cue on a left‐to‐right oriented representational continuum add to earlier behavioral demonstrations of SNAs in developmental populations who lack reading/writing and counting experience (e.g., Bulf et al., 2016; de Hevia, Veggiotti, et al., 2017; de Hevia et al., 2014; Di Giorgio et al., 2019), opening a window to the identification of early neural markers of atypical developmental trajectories as well as early interventions to support infant learning in the numerical domain.

Like prior infant studies, the current investigation is neutral with respect to the fundamental question of the origin of SNAs. Both biological constraints and early cultural experience have been proposed to be at the roots of this phenomenon. Examples of biological constraints include early temporal asymmetries in hemispheric maturation resulting in the dominance of the left over the right visual hemispace (e.g., Dubois et al., 2008), asymmetries in infants' oculomotor scanning behavior favoring the deployment of visuospatial attention along the horizontal plane (e.g., Van Renswoude, Johnson, Raijmakers, & Visser, 2016), a processing advantage for looming/approaching over zooming/retracting stimuli resulting in enhanced processing of increasing over decreasing magnitudes (de Hevia, Addabbo, et al., 2017; Macchi Cassia, Picozzi, Girelli, & de Hevia, 2012), and hemispheric asymmetry in brain sensitivity to the spatial frequency content of visual stimuli resulting in lateralized responses to increments and decrements in nonsymbolic numerosity (Felisatti, Aagten‐Murphy, Laubrock, Shaki, & Fischer, 2020).

All these biologically determined biases are thought to regulate somehow the asymmetrical exploration of space on which nonsymbolic SNAs is grounded. However, early implicit, directionally relevant experience provided to infants by caregivers and other adults have also been claimed to contribute to the early establishment of SNAs (see discussion in Nuerk, Moeller, Klein, Willmes, & Fischer, 2015), and more generally, of directional biases in the allocation of visuospatial attention (see discussion in Bulf, de Hevia, Gariboldi, & Cassia, 2017).

Reading habits are thought to play an important role in the emergence of cultural differences in visuospatial abilities (e.g., Rashidi‐Ranjbar, Goudarzvand, Jahangiri, Brugger, & Loetscher, 2014), as well as in SNAs (see Göbel et al., 2011), but there is now growing interest in how cultural experience may affect how infants represent numbers and other ordered information onto space even before entering formal education (e.g., Göbel et al., 2018; McCrink, Caldera, & Shaki, 2018; Patro, Fischer, Nuerk, & Cress, 2016; Patro, Nuerk, & Cress, 2016). Infants are exposed to a variety of spatially relevant culturally‐driven routines from the very onset of postnatal life, as adult caregivers structure the physical environment for their children in many different ways, including gazing, touching and pointing to items in space during dyadic and triadic interactions, play sessions and share book reading. Throughout these activities, adults ultimately influence how infants deploy attention to explore the external space.

Importantly, biological biases and cultural factors do not play a mutually exclusive role in shaping the tendency to associate numbers with spatial positions: From early on and across the life span, cultural experience could either strengthen a pre‐determined propensity to asymmetrically explore space or counteract it, eventually giving rise to the reported culturally dependent strategies to represent number (Göbel et al., 2011) and other ordered information (e.g., Guida et al., 2018). While this study is noninformative on the contribution of biology and culture to the observed neural markers of SNAs in 9‐month‐old infants, future research may explore this question by testing infants raised in cultures with right‐to‐left oriented (i.e., Eastern cultures) or mixed (i.e., Japanese; see Macchi Cassia et al., 2020 for relevant evidence in 7‐month‐old infants) reading/writing habits, which likely affect the quantity and quality of directional experience infants have access to.
